# CC-Chemokine CCL15 Expression and Possible Implications for the Pathogenesis of IgE-Related Severe Asthma

**DOI:** 10.1155/2012/475253

**Published:** 2012-10-31

**Authors:** Yasuo Shimizu, Kunio Dobashi

**Affiliations:** ^1^Department of Pulmonary Medicine, Maebashi Red Cross Hospital, 3-21-36 Asahi-Cho, Gunma, Maebashi 371-0014, Japan; ^2^Department of Medicine and Molecular Science, Gunma University Graduate School of Medicine, 3-39-15 Showa-Machi, Gunma, Maebashi 371-8511, Japan; ^3^Gunma University School of Health Sciences, 3-39-15 Showa-Machi, Gunma, Maebashi 371-8511, Japan

## Abstract

Airway inflammation is accompanied by infiltration of inflammatory cells and an abnormal response of airway smooth muscle. These cells secrete chemokines and express the cell surface chemokine receptors that play an important role in the migration and degranulation of inflammatory cells. Omalizumab is a monoclonal antibody directed against immunoglobulin E, and its blocking of IgE signaling not only reduces inflammatory cell infiltration mediated by the Th2 immune response but also inhibits other immune responses. The chemokine CCL15 is influenced by omalizumab, and the source of CCL15 has been reported to be airway smooth muscle cells and basophils. CCL15 binds to its receptor CCR1, which has been reported to be expressed by various inflammatory cells and also by airway smooth muscle cells. Therefore, CCL15/CCR1 signaling could be a target for the treatment of asthma. We review the role of CCL15 in the pathogenesis of asthma and also discuss the influence of IgE-mediated immunomodulation via CCL15 and its receptor CCR1.

## 1. Introduction

Chemokines play an important role in the accumulation of inflammatory cells. They belong to a superfamily of small (6–14 kDa) proteins that regulate trafficking in various cells [1]. The C-C motif chemokine ligand 15 (CCL15) is a member of the macrophage inflammatory protein-1 family of chemokines, and its gene is located on 17q11.2. The genetic sequence of CCL15 is similar to that of C-C motif chemokine ligand 5 (CCL5) which is known as regulated on activation normal T cell expressed and secreted (RANTES) and C-C motif chemokine ligand 3 (CCL3), which is named macrophage inflammatory protein-1*α* (MIP-1*α*). The CCL15 gene has four exons and three introns. CCL15 has also been variously termed macrophage inhibitory protein-5 (MIP-5), leukotactin-1 (Lkn-1), and human C-C chemokine 2 (HCC-2) [2–4]. CCL15 binds to two receptors known as CCR1 and CCR3, but it has a higher affinity for the former [2, 5, 6].

Although other chemokines, such as CCL3L1 and CCL5, have already been examined in detail to assess their role in asthma, little is known about the possible influence of CCL15 on asthma. Recently, the serum level of CCL15 was found to be elevated in patients with severe asthma and it was shown to be reduced by omalizumab, a humanized anti-IgE antibody [7]. In addition, airway smooth muscle cells (ASMCs) have been shown to produce CCL15 in vitro, and these cells express CCR1 in asthma patients [8, 9]. This paper reviews the role of CCL15 in the pathogenesis of asthma and also discusses the influence of IgE-mediated immunomodulation via CCL15 and its receptor CCR1.

### 1.1. CCL15 Expressions and Inflammatory Cells

CCL15 mRNA expression is abundant in the heart and skeletal muscle, and it is also detectable in the placenta, liver, pancreas, adrenal gland, bone marrow, colon, small intestine, lung, trachea, and ASMC [4, 8, 10]. Among the various inflammatory cells, CCL15 mRNA expression has been observed in human lung leukocytes, basophils, and alveolar macrophages, but no expression has been found in lymphocytes, neutrophils, monocytes, lung dendritic cells, or endothelial cell, fibroblast, and leukemia cell lines [2, 8].

The major role of CCL15 as a chemoattractant (similar to CCL3L1) is mediated via its receptor. CCL15 elicits a transient increase of intracellular calcium [Ca^2+^]i in isolated human blood monocytes and eosinophils, but it has little effect on lymphocytes and neutrophils [4]. In agreement with these findings, CCL15 has a chemotactic effect on human blood monocytes and eosinophils, but little effect on lymphocytes [4]. Neutrophils isolated from the peripheral blood showed a transient increase of [Ca^2+^]i when exposed to CCL15, but they did not show a chemotactic response [4]. However, another study showed that CCR1, which is the receptor for CCL15, is expressed by neutrophils isolated from peripheral blood, and CCL15 has a high binding capacity for CCR1 and induces chemotaxis of human neutrophils. CCL15 elicits a stronger neutrophil response compared with CCL3, while neutrophils from CCR1 knockout mice fail to respond to this chemokine [11]. Thus, CCL15 plays a role in chemotaxis, but its effects on inflammatory cells are not fully understood.

### 1.2. CCL15 Receptors and Intracellular Signaling

CCL15 binds to the cell surface receptors CCR1 and CCR3. The genes for CCR1 and CCR3 form a cluster on chromosome 3p, and these receptors belong to the C-C chemokine receptor family, which are seven transmembrane proteins similar to G-coupled protein receptors [12–14]. CCR1 is expressed by monocytes, macrophages, dendritic cells, T cells, B cells, as well as at a low level on mast cells, eosinophils, neutrophils, and ASMC [2, 14–17]. CCL15 induces G protein (Gi/Go) signal transduction, increases phospholipase C (PLC) activity, increases protein kinase C*δ* (PKC*δ*) activity, and activates the transcription factor nuclear factor-kappa B (NF-*κ*B), resulting in chemotaxis or protein synthesis or degranulation [5, 18]. The kinase activity of mitogen-activated protein (MAP) kinases such as extracellular signal-regulated kinase-1/2 (ERK1/2) and p38 contributes to CCL15-induced chemotaxis [19]. CCR3 is constitutively expressed at a high level on eosinophils, and is also expressed on basophils, mast cells, T cells (Th2), keratinocytes, and ASMC [14, 20]. Among the ligands of CCR3, the role of CCL3, CCL5, and CCL11 (eotaxin-1) in allergic inflammation has been extensively examined, but little is known about CCL15-induced CCR3 signal transduction. CCL11 induces the signal transduction pathway for G proteins (G*βγ*/G*α*i), increases Ras activity, increases phosphorylation of ERK2 and p38 MAP kinases, and promotes chemotaxis, cell differentiation, and protein synthesis or release by degranulation [21–23].

CCL15 binds to both CCR1 and CCR3, but it has a lower affinity for the latter receptor [2, 5, 6]. CCR1 was equally expressed by Th1 and Th2 cells in human cord blood lymphocytes [24]. CCR1 knockout mice have smaller experimental lung granulomas, which is related to increased interferon-*γ* (IFN-*γ*) production (Th1) with decreased production of interleukin-4 (IL-4) (Th2) in pulmonary lymph node cells. These results suggested that CCR1 not only influences the inflamatory response through a direct effect on leukocyte chemotaxis, but also by modulating the Th1 or Th2 cytokine balance [25]. Thus, signaling via CCR1 plays a role in the modulation of inflammation.

### 1.3. Expression of CCL15 and Its Receptor (CCR1) by Airway Smooth Muscle Cells and Basophils

Elevation of CCL15 protein levels had been reported in patients with various pulmonary diseases. CCL15 was elevated in the bronchoalveolar lavage fluid (BALF) obtained from patients with stage III sarcoidosis [26] and in peripheral blood from patients with severe persistent asthma, while anti-IgE antibody therapy reduced the CCL15 level of severe asthma patients [7]. Treatment of nonsmall cell lung cancer reduced the plasma level of CCL15 protein, and this was thought to be related to the influence of CCL15 on angiogenesis [27]. A recent study showed that CCL15 protein was elevated in the supernatant of cultured human airway smooth muscle cells (ASMCs) after stimulation with tumor necrosis factor-*α* (TNF-*α*), while the level was synergistically enhanced by adding IFN-*γ* (the synergy of TNF-*α* with IFN-*γ* was NF-*κ*B dependent). Expression of CCL15 mRNA was elevated in bronchial biopsy specimens from patients with moderate-to-severe asthma, and its level was higher in moderate asthma [8]. Elevation of CCL15 mRNA levels had been reported in human lung leukocytes and alveolar macrophages [2, 8], while basophils are also a source of CCL15. CCL15 protein production was observed when basophils isolated from the peripheral blood of asthma patients and nonasthmatic control subjects were stimulated with IL-3 [28]. Biopsy of the airways has demonstrated elevated expression of CCR1 mRNA in mild-to-severe asthma, and CCR1 expression in ASMC has been revealed by immunohistochemistry [9]. Basophils also express CCR1 in allergic responses [14, 29].

Thus, ASMC and basophils express the CCR1, and CCL15 has a high binding affinity for this receptor. These findings suggest that ASMC and basophils are important sources of CCL15, which might have a role in asthma and contribute to the severity and persistence of this condition through targeting its receptors (especially CCR1) in an autocrine manner.

### 1.4. Interaction of CCL15/CCR1 with Immunoglobulin E in Asthma

ASMCs not only express CCR1 and CCR3, but also express high-affinity (Fc*ε*RI) and low-affinity (Fc*ε*RII) IgE-Fc receptors. It has been reported that IgE induces abnormal smooth muscle contraction, while sensitization of ASMC to IgE elicits the sequential autocrine release of IL-4, IL-5, and IL-13, but not IFN-*γ* [30–32]. IgE cross-linking also induces the production of IL-6, IL-8, and TNF-*α* via ERK1/2 and p38 MAP kinases in ASMC isolated from the bronchial tissue of asthma patients, while cytokine production is inhibited by the anti-IgE antibody omalizumab [33]. Moreover, CCL15 protein is produced by ASMC after stimulation with TNF-*α*, and its production was synergistically enhanced by IFN-*γ* [8].

**Figure 1:**
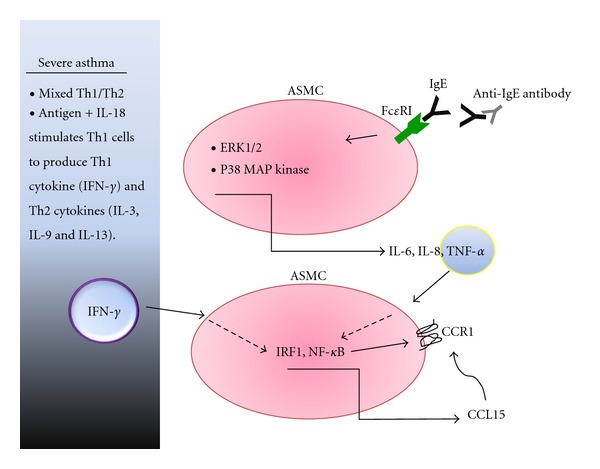
CCL15/CCR1-mediated inflammatory response associated with IgE in ASMCs from an asthma patient. IgE-stimulated ASMC produce IL-6, IL-8, and TNF-*α* via the activation of extracellularly regulated MAP kinase-1/2 (ERK1/2) and p38 MAP kinase. TNF-*α* also plays a role in CCL15 production by synergistically enhancing the effect of IFN-*γ* via interferon regulatory factor-1 (IRF-1) and NF-*κ*B. IFN-*γ* production is abundant in patients with mixed Th1/Th2 or Th1 asthma. TNF-*α* and IFN-*γ* upregulate CCR1 expression by ASMC, while binding of CCL15 to CCR1 might influence the severity and persistence of asthma.

The serum level of IFN-*γ* is related to the decline of FEV1 in asthma, and IFN-*γ* expression in the airway wall is higher in severe asthma than moderate asthma. IFN-*γ* mediates Th1 immune responses, while airway inflammation is a Th2 immune response that involves IL-4 and IL-13. However, the Th2 immune response is not essential in severe asthma and IFN-*γ* could be involved [34, 35]. Asthma is thus becoming recognized as a heterogenous disorder that presents a mixed Th1/Th2 phenotype with a contribution from Th17 (IL-17) cells [36, 37]. Th1 cells stimulated by antigens and IL-18 produce IFN-*γ* (a Th1 cytokine) as well as IL-9 and IL-13 (Th2 cytokines) [38, 39]. Based on these reports, the mechanism of IgE-mediated CCL15 production could be as follows. IgE stimulates ASMC to produce IL-6, IL-8, and TNF-*α*, after which TNF-*α* promotes CCL15 production by ASMC with a synergistic enhancement of this effect in the presence of IFN-*γ* (Figure 1).

TNF-*α* and IFN-*γ* upregulate CCR1 expression by ASMC, so binding of CCL15 to CCR1 might contribute to the severity and pesistence of asthma. Thus, IgE may indirectly modulate the CCL15/CCR1 axis in ASMC, while inhibition of these mechanisms by omalizumab might contribute to a reduction of CCL15 in patients with severe persistent asthma [7].

As described above, basophils are a source of CCL15, and these cells express CCR1, CCR3, and Fc*ε*RI. Although omalizumab therapy reduces Fc*ε*RI-mediated production of IL-4, IL-13, and IL-8 by basophils, CCL15 mRNA expression is not altered by Fc*ε*RI cross-linking [28]. The mechanism that regulates CCL15 production by basophils in response to IgE signaling remains unknown.

**Figure 2:**
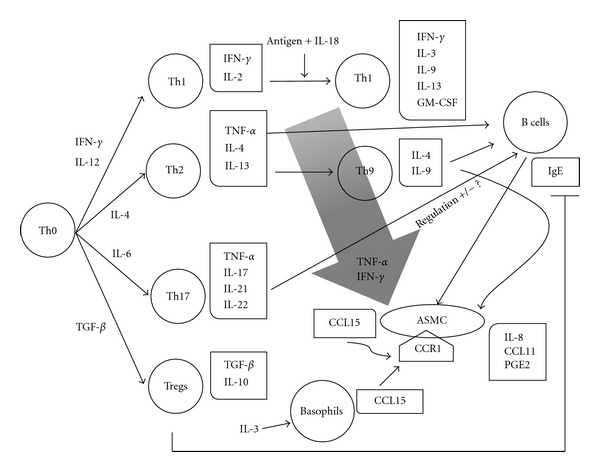
CD4+ Th subsets in asthma and the potential role of CCL15 production by ASMC. Mixed Th1 cells produce both Th1 and Th2 cytokines (IFN-*γ*, IL-3, IL-9, IL-13, GM-CSF) when stimulated by exposure to an antigen plus IL-18. Th2 and Th17 cells secrete TNF-*α*, while Th9 cells are differentiated from Th2 cells secrete IL-9, which promotes IgE production by B cells and the production of IL-8, CCL11, and prostaglandin E_2_ (PGE_2_) by ASMC. IgE promotes the inflammatory phenotype of ASMC, and these cells produce CCL15 when stimulated with both TNF-*α* and IFN-*γ*. IL-21 from Th17 cells has been suggested to regulate IgE production by B cells, and Tregs have an inhibitory effect on it.

It has been reported that Th1 cells not only produce Th1 cytokines, but also Th2 cytokines (IFN-*γ*, IL-3, IL-9, IL-13, and granulocyte-macrophage colony-stimulating factor, GM-CSF) [38, 39], and novel CD4+ subsets that include Th17 cells, Th9 cells, and regulator T cells (Tregs) have emerged as being involved in the pathogenesis of asthma (Figure 2). Th17 cells differentiate from Th0 cells in response to interleukin-6 (IL-6), and these cells produce interleukin-17 (IL-17), interleukin-21 (IL-21), and interleukin-22 (IL-22) [40]. IL-17 attracts neutrophils [41], while IL-21 influences IgE production by B cells [42].

Some Th2 cells switch to Th9 cells after stimulation by transforming growth factor-*β* (TGF-*β*) and IL-4 [43]. Th9 cells secrete IL-4 and IL-9, which enhance IgE production by B cells, and IL-9 also promotes the production of IL-8, CCL11, and prostaglandin E_2_ (PGE_2_) by ASMC [44]. Tregs are induced by TGF-*β*, and these cells secrete interleukin-10 (IL-10) and TGF-*β*. Tregs have an inhibitory effect on Th2 cells and also inhibit IgE production by B cells [45]. Although Th17 cells, Th9 cells, and Tregs may be involved in IgE-mediated asthma and seem to have an influence on ASMC, the associations of these Th subsets with the CCL15/CCR1 axis has not been explored in relation to the pathogenesis of asthma.

Blocking of IgE signaling also has effects on leukotrienes and prostaglandins. Omalizumab decreases the circulating levels of several leukotrienes (C4, D4, and E4) in children with allergic rhinitis [46], while prostaglandin D_2_ levels in nasal lavage fluid from allergic rhinitis patients are reduced by omalizumab therapy [47]. These mediators are also important in asthma, but their relation with the CCL15/CCR1 axis remains unknown.

Omalizumab has been reported to be effective for food allergies, allergic rhinitis, atopic dermatitis, and urticaria caused by various triggers [48]. An asthma patient on high-dose beclomethasone (>800 *μ*g/day) with a low forced expiratory volume (<65% of the predicted value) was reported to respond to omalizumab [49], but whether there is a different response depending on the trigger for asthma or a difference between moderate and severe asthma has not yet been determined. 

## 2. Other Effects of CCL15

**Figure 3:**
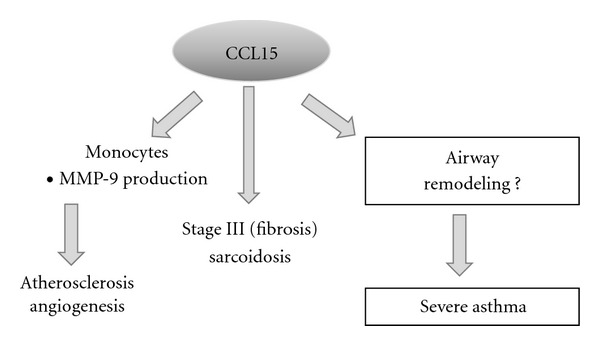
Other effects of CCL15. It has been shown that CCL15 has a role in atherosclerosis via macrophage activation, and that it promotes angiogenesis in lung cancer. CCL15 has also been suggested to show an association with the fibrotic stage of sarcoidosis, but its role in airway remodeling remains unknown.

Airway remodeling is involved in the pathogenesis of severe persistent asthma. Omalizumab therapy reduced airway wall thickening in patients with severe asthma, along with a reduction of the sputum eosinophil count [50]. CCL15 has also been reported to contribute to plaque instability during the progression of atherosclerosis by promoting the release of matrix metalloproteinase-9 from THP-1 cells [51]. Furthermore, CCL15 promotes angiogenesis in lung cancer [27]. Moreover, CCL15 is elevated in patients with advanced (stage III) sarcoidosis [26], as well as in patients with moderate-to-severe asthma [7, 8]. Thus, CCL15 may contribute to both chronic inflammation and airway remodeling (Figure 3).

### 2.1. Clinical Trials of Omalizumab Therapy for Asthma

There have been many studies investigating the efficacy of omalizumab for moderate-to-severe asthma. The Investigation of Omalizumab in severe Asthma TrEatment study (INNOVATE study) showed a decrease of exacerbations in patients with severe asthma and improvement of the asthma quality of life questionnaire (AQLQ) [52]. In addition, regular use of asthma medications was reduced in patients with severe asthma [53], and the use of rescue medications was also decreased in patients with moderate-to-severe asthma [54].

A large-scale prospective study of omalizumab is ongoing (The Epidemiologic study of Xolair (omalizumab): evaluating Clinical Effectiveness and Long-term Safety in patients with moderate-to-severe asthma (EXCELS study)) [55]. According to a recent report on 2-year data from this study, patients initiating omalizumab therapy experienced clinically relevant improvement, whereas established users of omalizumab maintained control of their asthma along with slight improvement or a similar outcome to that seen in nonusers of this agent [56]. A study of the effect of omalizumab on clinical improvement of asthma and inflamatory mediators showed a reduction in the release of cytokines (IL-4, Il-8, and IL-13) by basophils from omalizumab-treated patients, along with the reduction of IL-5 and IL-13 release in cocultures of plasmacytoid dendritic cells and T cells. These studies also suggested that IgE probably facilitates the presentation of allergens by dendritic cells in vivo and has an important role in regulating DC-dependent T cell cytokines during the effector phases of allergic disease [57].

## 3. Conclusion

CCL15 was reduced in asthma patients by omalizumab and ASMCs were considered to be the source of CCL15. Anti-IgE therapy with omalizumab improves asthma, and several possible mechanisms of immunomodulation by omalizumab have been reported. As omalizumab is used more widely, further effects of this agent may be discovered. Although the chief role of the CCL15/CCR1 axis has been considered to involve promoting the accumulation of inflammatory cells in the airways of asthma patients; CCL15 may also make a contribution to the severity of asthma and to airflow limitation via effects on ASMC. Thus, CCL15 could be a potential target for asthma therapy, although little is known about its contribution to the pathophysiology of this disease.
